# Müllerian Agenesis Masquerading as Secondary Amenorrhea

**DOI:** 10.1155/2018/6912351

**Published:** 2018-07-19

**Authors:** Gloria Tavera, Rina Lazebnik

**Affiliations:** ^1^Case Western Reserve University School of Medicine, Cleveland, OH, USA; ^2^Department of General Pediatrics and Adolescent Medicine, University Hospitals Cleveland Medical Center, Case Western Reserve University School of Medicine, Cleveland, OH, USA

## Abstract

The most common cause of primary amenorrhea is congenital malformation of the Müllerian ducts, including Müllerian agenesis, also known as Mayer–Rokitansky–Küster–Hauser syndrome (MRKH). Most general gynecologists and primary care physicians who see female adolescents will encounter MRKH in their careers. We present the case of an adolescent with MRKH who reported secondary, instead of primary amenorrhea. We discuss the subtleties of diagnosing MRKH, especially when patient history may not always be accurate. Because MRKH had not been included in the differential diagnosis for delayed menses, this patient was initially misdiagnosed. Delayed diagnosis of MRKH may harm patients by delaying assessment of concomitant renal, skeletal, hearing, and cardiac defects, which might otherwise impact the treatment plan.

## 1. Introduction

Müllerian agenesis, also called Mayer–Rokitansky–Küster–Hauser syndrome (MRKH). is a congenital malformation of the Müllerian ducts, resulting in an absent uterus and variable degrees of hypoplasia of the fallopian tubes, cervix, and first two-thirds of the vagina. It is the most common cause of primary amenorrhea, affecting approximately 1 in 4,500 females [1]. It results from an unknown combination of polygenic and environmental factors [2]. Women with MRKH have a 46 XX karyotype, functioning ovaries, developed external genitalia, and other secondary sex characteristics. Vaginal creation and assisted reproduction are possible, but women cannot carry a pregnancy, outside of the intervention of a uterine transplantation [3, 4]. Workup requires monitoring for possible concomitant renal, skeletal, hearing, and cardiac defects, as well as psychological counseling [5]. This report summarizes a diagnosis of MRKH made in the teenage years and highlights the presence of the diagnosis with a given history of secondary amenorrhea.

## 2. Case Presentation

A 15-year-old female was returning for follow-up after a 1-month medroxyprogesterone acetate challenge test. The patient had been seen at the clinic prior to age 10 and returned at age 14, reporting menarche at age 14. The patient returned at age 15 and reported that menstruation had started and stopped twice. Free testosterone was high (6.8 pg/mL), and polycystic ovary syndrome was suspected. This patient history may have deterred clinicians from initially including a differential diagnosis of MRKH. The patient was given the medroxyprogesterone acetate challenge test for suspected secondary amenorrhea and returned for follow-up, after 1 month. The patient had not menstruated after the medroxyprogesterone challenge test.

In a detailed sexual history, the patient reported being sexually active, including vaginal penetration and excluding anal penetration. At this visit, the patient reported continued amenorrhea, lower abdominal pain, and frequent urinary tract infections (UTIs).

Upon attempted collection of a genital swab specimen for sexually transmitted disease (STD) testing, labia minora and majora were present, but no opening to the vagina could be identified, such that the genital swab could not penetrate beyond a wall of pale pink, thin tissue, immediately past labia minora. Further physical examination of the genital tract, or insertion of a speculum, was not possible due to this abnormality. There were no masses in the abdomen, and urethral and rectal openings were intact and fully developed. Ultrasound confirmed the lack of a vaginal canal, and magnetic resonance imaging (MRI) confirmed the presence of a remnant uterus, consistent with a diagnosis of MRKH. The MRI also screened for possible concomitant defects.

MRI results confirmed a suspected diagnosis of MRKH with uterine aplasia. In the presumed location of the uterus, there was a longitudinal soft tissue plate measuring 2.5 × 1.4 centimeters. There was also no direct communication to the vulvar region. Bilateral ovaries were identified and demonstrated developed follicles. The presumed location of the vaginal canal was visualized with fluid and debris inside, which may have resulted from a lack of an opening to the vulva. MRI also revealed a mildly asymmetric and dysmorphic sacrum and L5 vertebral body.

Bilateral kidneys were present in the expected location. The left renal collecting system was duplicated, with mild hydronephrosis and hydroureter, extending to the level of the left common iliac artery. Both ureters on the left side were mildly dilated, down to the crossing of the iliac artery. More distally, the caliber of duplicate ureters was within normal limits. The right kidney was unremarkable but with a slightly prominent ureter.

The patient had noted hearing loss. A pure tone audiometry test demonstrated conductive hearing loss at low frequencies, in the right ear. However, auditory brainstem response testing of the inner ear (cochlea) and brain pathways for hearing were within normal limits.

The patient had a history of psychiatric diagnoses and was receiving pharmacological treatment (clonidine and methylphenidate) and counseling. Socioeconomic and familial challenges likely contributed to development and exacerbation of psychiatric and behavioral issues. In addition to facing poverty, the patient's mother was deaf and partially blind, and the patient served as caretaker.

When the diagnosis of MRKH was delivered and explained to the patient and her mother, the patient reported regular bladder and bowel function and continued amenorrhea. The patient also reported suicidal ideation, but reported major improvements in quality of life after taking residence in a behavioral health rehabilitation facility and maintaining regular psychiatric appointments and medications.

After explanation of the diagnosis using charts and diagrams, the mother and patient confirmed that they understood the implications on reproduction. The patient continued to affirm that she briefly menstruated at ages 14 and 15. When asked to elaborate, the patient explicitly reported 4 months of menstruation, followed by amenorrhea, followed by 2 more months of menstruation, then amenorrhea until present, now approaching her 16th birthday. The patient was referred to an adolescent obstetrician-gynecologist for consultation for possible vaginoplasty/vaginal creation.

## 3. Discussion

Current guidelines highlight the need for examination of internal genitalia in patients with menstrual disorders, younger than 21 years of age [6]. The patient reported lower abdominal pain and lack of regular menstrual cycles and had a history of frequent UTIs, all of which are indications for a pelvic examination [7]. Renal abnormalities were detected in this patient and are commonly associated with MRKH [3]. MRI also revealed a mildly asymmetric and dysmorphic sacrum and L5 vertebral body. The patient was previously diagnosed with sacral agenesis, with only 4 lumbar vertebrae, which is associated with MRKH syndrome [3, 8]. Hearing deficits are also associated with MRKH syndrome and were present in this patient [3]. Lastly, free testosterone remained high in this patient and is also associated with MRKH [3].

This case illustrates the importance of monitoring for MRKH in the young adolescent population, as it is a relatively common condition whose major indicator of primary amenorrhea may not always be accurately reported. Inaccurate reporting may occur due to confusion of rectal bleeding or external vaginal irritation with menses. Inaccurate reporting may also occur out of shame, misunderstanding of the questioning, or social or psychological complexities.

The fact that the patient continued to report secondary amenorrhea even after the discovery that she had a nonfunctioning uterus remains an interesting point of discussion. The patient did not report the symptom of primary amenorrhea that patients with MRKH usually report, and a pelvic exam had not been performed before the age of 15. The patient described the brief occurrence of menses-like bleeding and reported being sexually active, with vaginal penetration. The patient had been seen in the clinic before and had reported secondary amenorrhea. At that time, no pelvic exam was performed and medroxyprogesterone was prescribed as a challenge test for secondary amenorrhea. If a pelvic exam had been attempted when the patient had first reported secondary amenorrhea, the diagnosis of MRKH could have been achieved more quickly. In this case, the pelvic exam could proceed no further than visual inspection, since insertion of a speculum was not possible, nor was a bimanual exam. Any report of a menstrual disorder in an individual younger than 21 years of age merits a pelvic exam, following American Congress of Obstetricians and Gynecologists (ACOG) indications [6].

A delayed diagnosis of MRKH may harm patients by delaying assessment, monitoring, and intervention for likely concomitant renal, skeletal, hearing, or cardiac defects. This might impact the treatment plan, when considering a syndrome of congenital malformations. Delayed diagnosis can also lead to missed opportunities to explain to patients the origin of their amenorrhea, which impacts family planning, and to follow up with psychological counseling. Encouraging annual visits to the pediatrician is key to preventing delayed diagnosis.

This case highlights the importance of keeping MRKH syndrome in mind as a differential diagnosis, when the sexual history includes any report of amenorrhea. Indicators for a pelvic exam in patients with MRKH syndrome include amenorrhea and may include frequent urinary tract symptoms and lower abdominal pain, all of which were present in this patient. Building a relationship of trust and good communication with the patient is important, prior to performing a pelvic exam. The time and effort put into the relationship may be key to uncovering important physical exam findings that add to the patient's reported sexual history.
